# Modeling Personalized Adjuvant TreaTment in EaRly stage coloN cancer (PATTERN)

**DOI:** 10.1007/s10198-020-01199-4

**Published:** 2020-05-26

**Authors:** Gabrielle Jongeneel, Marjolein J. E. Greuter, Felice N. van Erning, Miriam Koopman, Jan P. Medema, Raju Kandimalla, Ajay Goel, Luis Bujanda, Gerrit A. Meijer, Remond J. A. Fijneman, Martijn G. H. van Oijen, Jan Ijzermans, Cornelis J. A. Punt, Geraldine R. Vink, Veerle M. H. Coupé

**Affiliations:** 1 Department of Epidemiology and Biostatistics, Amsterdam UMC, VU University, MF F-wing, PO Box 7057, 1007 MB Amsterdam, The Netherlands; 2 Department of Research, Netherlands Comprehensive Cancer Organization (IKNL), Utrecht, The Netherlands; 3 Department of Medical Oncology, University Medical Center Utrecht, Utrecht University, Utrecht, The Netherlands; 4 Department of Radiotherapy, Amsterdam UMC, University of Amsterdam, Amsterdam, The Netherlands; 5 Center for Gastrointestinal Research, Center for Translational Genomics and Oncology, Baylor Scott & White Research Institute and Charles A. Sammons Cancer Center, Baylor University Medical Center, Dallas, TX USA; 6 Instituto Biodonostia, Department of Gastroenterology Centro de Investigación Biomédica en Red de Enfermedades Hepáticas y Digestivas (CIBERehd), Universidad del País Vasco (UPV/EHU), San Sebastián, Spain; 7 Department of Pathology, The Netherlands Cancer Institute, Amsterdam, The Netherlands; 8 Department of Medical Oncology, Amsterdam UMC, University of Amsterdam, Amsterdam, The Netherlands; 9 Department of General Surgery, Erasmus MC University Medical Center, Rotterdam, The Netherlands

**Keywords:** Colon cancer, Adjuvant chemotherapy, Markov cohort model, Survival analysis, D61, I19

## Abstract

**Aim:**

To develop a decision model for the population-level evaluation of strategies to improve the selection of stage II colon cancer (CC) patients who benefit from adjuvant chemotherapy.

**Methods:**

A Markov cohort model with a one-month cycle length and a lifelong time horizon was developed. Five health states were included; diagnosis, 90-day mortality, death other causes, recurrence and CC death. Data from the Netherlands Cancer Registry were used to parameterize the model. Transition probabilities were estimated using parametric survival models including relevant clinical and pathological covariates. Subsequently, biomarker status was implemented using external data. Treatment effect was incorporated using pooled trial data. Model development, data sources used, parameter estimation, and internal and external validation are described in detail. To illustrate the use of the model, three example strategies were evaluated in which allocation of treatment was based on (A) 100% adherence to the Dutch guidelines, (B) observed adherence to guideline recommendations and (C) a biomarker-driven strategy.

**Results:**

Overall, the model showed good internal and external validity. Age, tumor growth, tumor sidedness, evaluated lymph nodes, and biomarker status were included as covariates. For the example strategies, the model predicted 83, 87 and 77 CC deaths after 5 years in a cohort of 1000 patients for strategies A, B and C, respectively.

**Conclusion:**

This model can be used to evaluate strategies for the allocation of adjuvant chemotherapy in stage II CC patients. In future studies, the model will be used to estimate population-level long-term health gain and cost-effectiveness of biomarker-based selection strategies.

**Electronic supplementary material:**

The online version of this article (10.1007/s10198-020-01199-4) contains supplementary material, which is available to authorized users.

## Background

With around 10,500 new cases and 4000 deaths in 2016, colon cancer is a major disease in the Netherlands leading to a substantial burden for patients, health care and society [1]. Over 25% of newly diagnosed colon cancer patients have stage II disease and it is likely that this will increase due to the recently initiated population-based colorectal cancer screening program [2, 3].

In stage II colon cancer, surgical resection is the curative treatment option of choice, followed by adjuvant therapy in a subgroup of patients. The benefit of adjuvant chemotherapy after surgical resection remains a matter of debate. Adjuvant chemotherapy is often recommended for stage II patients with a high risk of recurrence. However, there is no consensus on which factors predict the benefit from adjuvant chemotherapy. The correct identification of prognostic and predictive parameters is essential to optimize survival without inducing the harms of overtreatment in patients who will not benefit.

Until 2014, high-risk stage II patients were identified using clinical and pathological factors, i.e., pT4 stage (i.e., tumor growth), < 10 lymph nodes evaluated, perforation, vascular invasion, perineural invasion, and a high degree of differentiation [4–6]. In response to new findings, the Dutch guidelines were updated in 2014 with microsatellite stability (MSS) status in addition to the high-risk features mentioned above. In 2018, the Dutch association for medical oncology (NVMO) indicated that only pT4 and MSS status should be considered in the decision to allocate adjuvant chemotherapy in stage II colon cancer patients [7–10]. However, adherence to these guideline recommendations is low. In addition to substantiated deviations from the guideline recommendations, possible explanations for low adherence are unfamiliarity with this guideline, differences in expert opinions, and the clinical condition of the patient [11]. Presumably, increased compliance due to more awareness of the guideline recommendations could lead to health gains.

Additional molecular markers may enable improved patient selection for adjuvant chemotherapy as a number of specific aberrations, such as BRAF and KRAS mutations, are associated with prognosis [12–18]. To illustrate, MSI positive stage II and III patients with double wild-type cancers had a 5-year cancer-specific survival of 93% (95% CI 84–100%); while, patients with cancers harboring mutations in either BRAF or KRAS had a 5-year cancer-specific survival of 76% (95% CI 67–85%) [15]. The potential value of using these subtypes to inform adjuvant chemotherapy selection in colon cancer patients is acknowledged by several studies [12, 14].

Despite these developments in the field of colon cancer, the cost-effectiveness of different (biomarker-based) strategies for selecting high-risk stage II colon cancer patients has not been assessed so far. To address this issue, we developed the Personalized Adjuvant TreaTment in EaRly stage coloN cancer (PATTERN) model to synthesize evidence on different aspects of the decision problem, such as disease-free survival, overall survival, biomarker status, treatment effect, health utilities, and costs, from different sources in one coherent framework. This model can be used to evaluate the population-level cost-effectiveness of biomarker-based selection strategies for high-risk stage II colon cancer patients. Model structure, model assumptions, data sources, quantification and internal and external validation of model predictions are presented in this paper. As an example of the application of the model, the NVMO guideline and a hypothetical biomarker-driven strategy are compared with observed adherence to guideline recommendations based on data of the Netherlands cancer registry (NCR).

## Methods

### Description of the Markov cohort model

**Fig. 1:**
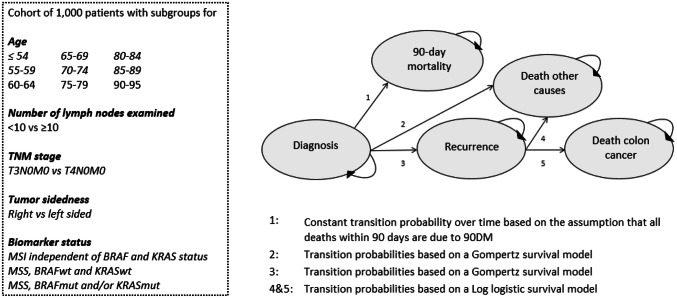
Structure of the Personalized Adjuvant TreaTment in EaRly stage coloN cancer (PATTERN) model

The Personalized Adjuvant TreaTment in EaRly stage coloN cancer (PATTERN) model is a deterministic Markov cohort model that simulates the disease progression of stage II colon cancer patients from the moment of diagnosis until death. A flowchart of the model is shown in Fig. 1. A Markov model describes a sequence of possible events in which the probability of a subsequent event depends solely on the state currently attained. This means that the time spent in a health state or the specific health states that are visited has no effect on the probability of a future transition. This is commonly referred to as the no-memory property. To be able to take time-dependent hazards into account, such as the hazards for the transition from recurrence to death, we incorporated tunnel states in the Markov model [19]. We opted for a cohort approach instead of a micro-simulation approach to increase the computational speed, such that the probabilistic sensitivity analysis is possible for future cost-effectiveness analyses. This choice for this approach seems counter intuitive for a relatively complex decision model, but it was possible by replicating the cohort model for each possible subgroup (defined on the basis of the included covariates).

We used a 1-month cycle length in the model. The model consists of 5 clinical states: diagnosis, 90-day mortality (90DM), recurrence, death due to other causes than colon cancer (DOC) and death of colon cancer (DCC). All patients start in the state ‘diagnosis’, and are treated surgically after pathological diagnosis. Furthermore, this state contains the option for patients to undergo or not undergo adjuvant chemotherapy. From diagnosis, patients may die within 90 days after resection due to complications after surgery or poor clinical condition (DIAG-90DM), they may die from DOC (DIAG-DOC) or they may develop a recurrence (DIAG-REC). After transitioning to ‘recurrence’, patients are again at risk of DOC (REC-DOC) and DCC (REC-DCC). In the model, probabilities for the transitions DIAG-90DM, DIAG-DOC and DIAG-REC were considered as competing events.

### Data used for parameter estimation

**Fig. 2:**
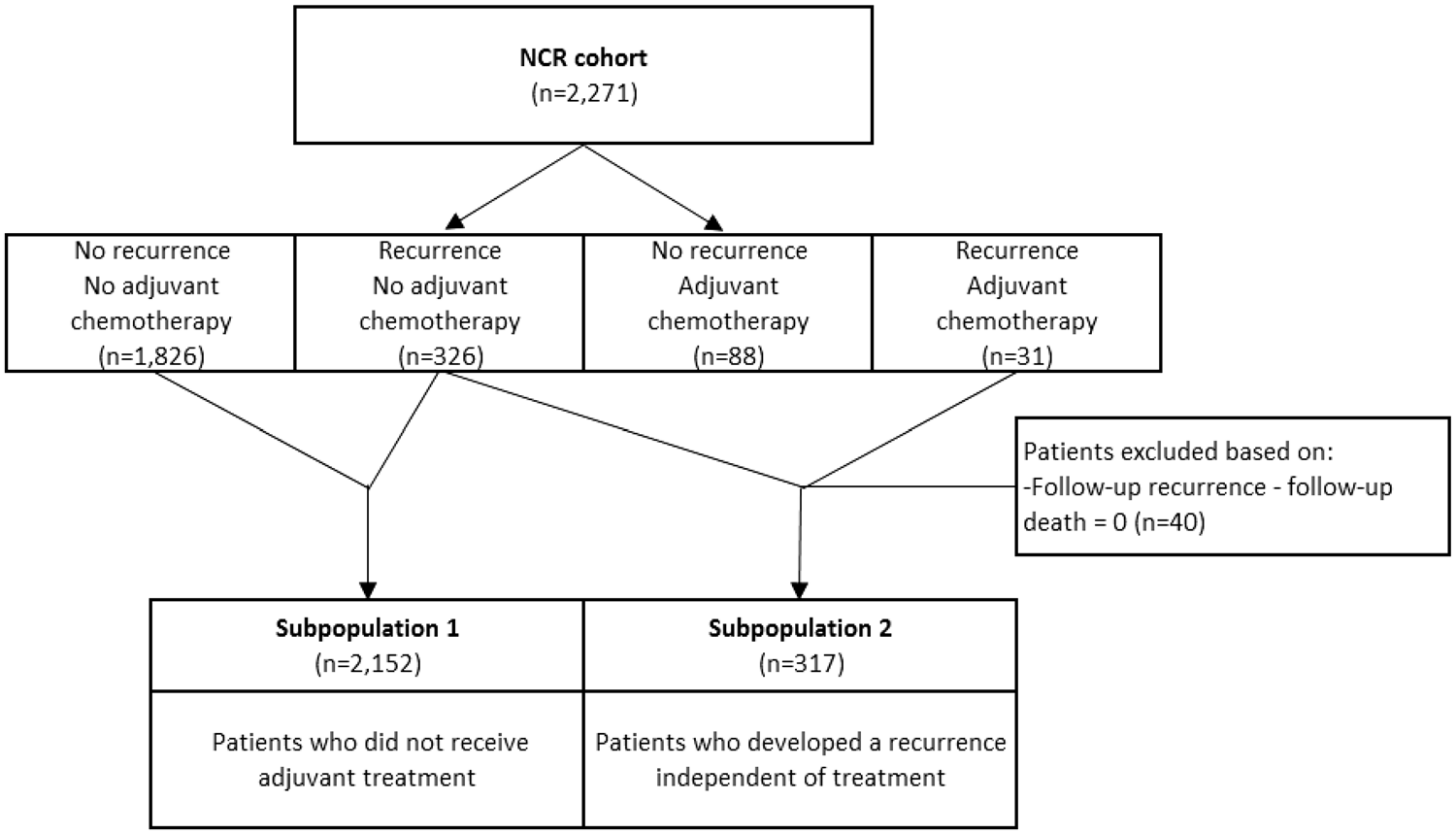
Flowchart of the 2002–2008 NCR data

Model quantification was based on data from the NCR. Recurrence follow-up data are not collected by default in the NCR. Therefore, we used a selection of 2271 patients from the nationwide registry for which this information was collected. The dataset consists of patients diagnosed with stage II colon cancer between 2002 and 2008. Patient and tumor characteristics, time to recurrence and time to death were collected. To estimate transition parameters using this dataset, we defined two partly overlapping subpopulations; (1) patients who underwent surgery and did not receive adjuvant chemotherapy, with or without a recurrence in the follow-up (*n* = 2152) and (2) patients who underwent surgery and developed a recurrence, with or without prior adjuvant chemotherapy (*n* = 317) (Fig. 2).

Biomarker data were not collected in the NCR; therefore, we used data of three external cohorts to include biomarker status. The following three cohorts were used; (1) a cohort from the MicroArray and proteomics Technologies to analyze Colorectal cancer and Hepatic metastases (MATCH) study [20], (2) a cohort obtained through the Baylor Scott and White Research Institute and Charles A Sammons Cancer Center (Texas cohort) [21] and (3) a cohort obtained through Instituto Biodonostia, Universidad del País Vasco, Centro de Investigación Biomédica en Red de Enfermedades Hepaticas y Digestivas (DONOSTIA cohort). These cohorts consisted of 105, 133 and 96 stage II colon cancer patients, respectively, and provided data on patient and tumor characteristics as well as biomarker status for MSS, BRAF (MATCH only) and KRAS (MATCH only).

### Parametrization of the model

For DIAG-90DM, we estimated a time-independent transition probability using the whole NCR population (*n* = 2271), based on the assumption that all observed deaths within 90 days are due to surgical complications or comorbidities [22, 23]. For all other transitions, the model was parametrized by parametric survival models including relevant covariates. The prognostic value of the following covariates was tested in our survival models: pT stage, tumor sidedness, differentiation grade, number of evaluated lymph nodes and age. The covariates were selected based on clinical relevance and data availability.

Transitions DIAG-DOC and DIAG-REC were estimated in subpopulation 1, in which patients with adjuvant chemotherapy were excluded. Because we aimed to include the impact of treatment on recurrence in the model on the basis of high-level evidence from randomized trials, we used the untreated patients from the NCR to estimate these two transitions in the absence of treatment [24]. Transitions REC-DOC and REC-DCC were estimated in subpopulation 2, in which only patients were included who developed a recurrence. We assumed that the administration of adjuvant chemotherapy after surgery does not influence mortality in patients with a recurrence. Therefore, patients treated with adjuvant chemotherapy were not excluded in subpopulation 2.

To estimate the parametric survival models, we tested for each transition four commonly used parametric survival distributions in health economic modeling (Weibull, Log normal, Log logistic and Gompertz) [25]. The choice for a distribution was based on the lowest Akaike’s information criterion (AIC) in combination with strict visual inspection in a model without covariates [25, 26]. After selecting the best fitting distribution, the prognostic value of the abovementioned covariates was assessed using a forward selection procedure. The added covariate was tested for significance using the Wald statistic, considering a two-sided *p* value of < 0.157 as statistically significant. Subsequently, the variable selection was extensively discussed with clinical experts, to ensure a conceptually valid inclusion of covariates. As a final check, the four parametric model distributions were again compared based on the lowest AIC in the models including the relevant covariates, which were selected in the previous step. This final check was conducted to confirm that the distribution selected in step 1 was still the best fitting distribution after covariate inclusion. It should be noted that the best fitting distribution did not change after covariate inclusion for any of the transitions.

In the dataset used for model parametrization, only overall survival was reported. That is, no distinction was made between DOC and DCC. Making this distinction is necessary to estimate the impact of improved selection strategies for adjuvant chemotherapy administration on the number of deaths due to colon cancer. To quantify the transition REC-DEATH (REC-DOC + REC-DCC), we selected the patients who developed a recurrence (subpopulation 2). This subpopulation was used to estimate a survival model with time to death as the outcome. Subsequently, we differentiated between DOC and DCC by assuming that the probability of DOC is equal for patients with and without a recurrence. That is, we assumed the transition REC-DOC to be equal to the transition DIAG-DOC in which DOC was estimated in the population without recurrence. The remaining deaths were considered due to DCC. All survival models were estimated using the flexsurvreg package in Rstudio version 3.4.2 [27].

### Addition of biomarker status

Literature shows that MSS, BRAF and KRAS mutation status are associated with prognosis in stage II colon cancer patients and that these factors may enable improved patient selection for adjuvant chemotherapy [12–18]. Therefore, we distinguished three biomarker subgroups in the model; (1) microsatellite instable tumors (MSI), independent of BRAF and KRAS status, (2) microsatellite stable tumors (MSS) without a mutation for BRAF or KRAS (MSSdwt), and (3) MSS in combination with a mutation in BRAF and/or KRAS (MSSmut).

To incorporate biomarker status in the transition DIAG-REC in the PATTERN model, the survival model reflecting time to recurrence since diagnosis was adjusted by including a hazard ratio (HR) for each biomarker subgroup. It should be noted that we assumed the same effect of biomarker status in all subgroups included in the PATTERN model. The HR for the MSI subgroup was estimated using the MATCH, Texas and Donostia cohorts. The HRs for the MSSdwt and MSSmut subgroups were estimated using the MATCH cohort only, as in the Texas and Donostia cohorts, KRAS and BRAF mutation status was unknown. The HRs for the MSSdwt and MSSmut subgroups were estimated relative to the MSI subgroup. The HRs for the biomarker subgroups were both estimated in a Gompertz parametric survival model and were applied directly to the overall hazard predicted by the Gompertz parametric survival model for the transition DIAG-REC, in which the clinical features were included.

### Addition of treatment effect

The selection strategy determines which patients receive adjuvant chemotherapy after diagnosis. The incorporated treatment effect was not based on the NCR data due to confounding by indication in the dataset. We implemented a treatment effect for adjuvant chemotherapy based on a meta-analysis of 9 Randomized Clinical Trials (RCTs) evaluating the effectiveness of adjuvant therapy in stage II colon cancer patients, published between 1999 and 2011. An extensive description of the used procedure is given elsewhere [24]. In short, we systematically searched relevant trials which reported summary disease-free survival data. Second, we generated patient-level data from the reported summary of survival data in the included trials using the method described by Hoyle and Henly [28]. Patient-level data of 4489 patients (events: 853) were generated from seven trials which compared fluoropyrimidine monotherapy to no adjuvant treatment (population 1). Furthermore, patient-level data of 1587 patients (events: 341) were generated from two trials which compared FOLFOX to fluoropyrimidine monotherapy (population 2). In the first population, a HR for fluoropyrimidine monotherapy compared to no adjuvant treatment of 0.78 (0.68; 0.89) was estimated in a Gompertz parametric survival model. In the second population, a HR for FOLFOX compared to fluoropyrimidine of 0.94 (0.76; 1.16) was estimated in a Gompertz model. To calculate a HR for FOLFOX compared to no adjuvant chemotherapy, we multiplied the HRs estimated in population 1 and 2, which resulted in a HR of 0.73. We implemented treatment effect in the PATTERN model by adjusting the transition DIAG-REC. As data on treatment heterogeneity are lacking, potential differences in treatment effect between subgroups were not taken into account.

### Competing risk correction

For transitions DIAG-90DM, DIAG-DOC and DIAG-REC, parametric survival modeling allowed for the estimation of the cause-specific hazard rates, which means that competing events were treated as censored for the event of interest. Because 90DM, DOC and recurrence are mutually exclusive events in the Markov cohort model, a competing risk correction was required. This correction was conducted according to the cumulative incidence competing risk (CICR) method [29, 30], which has previously been applied in a health economic model [31].

In essence, the corrected cumulative risk by time t is an estimate of the risk of failure from a specific cause, acknowledging that the absolute risk of the event is lowered by the presence of other competing risks. The corrected instantaneous risk at time t to experience each one of the transitions DIAG-90DM, DIAG-DOC and DIAG-REC is calculated by multiplying the hazard to experience each specific event at time t multiplied by the cumulative chance to be free of any of the three events at *t* − 1. The competing risk correction was carried out in discrete time steps of one month, corresponding to the 1-monthly cycles used in the Markov model. Note that for transitions REC-DOC and REC-DCC, a competing risk correction was not necessary, because these transitions were estimated jointly in the same survival model due to limitations in the data.

### Internal validity of the PATTERN model

First, we evaluated the internal validity of the final parametric survival models by visual inspection. That is, we compared the predicted recurrence and survival rates with their 95% confidence intervals to the NCR data. Second, the model performance was evaluated with the Greenwood–D’Agostino–Nam test for model calibration, which is a modification of the Hosmer–Lemeshow statistic [32]. The test for model calibration was assessed by dividing the cohort into deciles based on the predicted risk at 36 months. Subsequently, predicted and observed risks were compared. Third, the discriminatory capacity of the parametric survival models was assessed using the Uno modification of the Harrel’s *c*-statistic, which is suitable for censored survival data [33, 34]. Finally, to show that the model simulations correspond well with the NCR data used for model development, we compared the model estimates for recurrence and overall survival rate with data estimates and the corresponding 95% confidence interval at 12, 24, 36, 48 and 60 months. This approach was conducted for the overall population, and for subgroups for age (< 70 and > 70), pT stage (pT3 and pT4), number of lymph nodes examined (< 10 and ≥ 10) and tumor sidedness (left and right).

### External validation of the PATTERN model

To evaluate the external validity of the PATTERN model, model predictions for recurrence and overall survival rate were compared to the observed data of the NCR 2015 cohort, which was not used for model development. The NCR 2015 dataset consists of 1214 stage II colon cancer patients who did not receive adjuvant treatment for whom 3-year follow-up data were available for recurrence and overall survival (Online Appendix Table 1). Patient subgroups in the model were weighed in accordance with the subgroup distribution in the 2015 cohort. The model-predicted number of recurrences and deaths was compared to the 2015 data at 12, 24 and 36 months for the overall population and subgroups for age (≤ 70 and > 70), pT stage (pT3 and pT4), number of lymph nodes examined (≤ 10 and > 10) and tumor sidedness (left and right). If the model predictions did not fit within the 95% confidence interval of the data for all 3 evaluated time points for a specific subgroup, the PATTERN model was updated by adjusting the regression coefficients for that subgroup.

In addition, the implemented treatment effect in the PATTERN model, which was based on external RCT data, was validated in a separate analysis. First, survival curves for recurrence and overall survival were constructed for the subset of patients from the 2002–2008 NCR dataset (*n* = 129) and the 2015 NCR dataset (*n* = 115) who received adjuvant treatment (Table 1 and Online Appendix Table 1). Second, we set up the PATTERN model to simulate a scenario in which patients receive adjuvant chemotherapy. Note that we weighed the subgroups in the model such that it reflected the subgroup distribution in the systemically treated population of cohort 2002–2008 and 2015. Finally, we visually assessed the agreement between the model predictions for recurrence and overall survival with the data estimates at 12, 24, 36, 48 and 60 months for cohorts 2002–2008 and at 12, 24 and 36 months for cohort 2015.

**Table 1:** Patient characteristics NCR cohort 2002–2008

Variable	Whole population^a^ (*n* = 2271)	Subpopulation 1^b^ (*n* = 2152)	Subpopulation 2^c^ (*n* = 317)	Adjuvant treated patients^d^ (*n* = 129)
Age (years)	70.7 (10.9)	71.5 (10.7)	70.2 (9.4)	61.3 (10.6)
Gender				
Male	1078 (47.5)	1020 (47.4)	145 (45.7)	62 (48.1)
Female	1193 (52.5)	1132 (52.6)	172 (54.3)	67 (51.9)
pT stage				
pT3	2007 (88.4)	1931 (89.8)	260 (82.0)	83 (64.3)
pT4	214 (9.4)	171 (7.9)	56 (17.7)	46 (35.7)
Unknown	5 (2.2)	50 (2.3)	1 (0.3)	
Evaluated lymph nodes				
< 10	1198 (52.8)	1123 (52.2)	194 (61.2)	81 (62.8)
≥ 10	946 (41.7)	906 (42.1)	104 (32.8)	44 (34.1)
Unknown	127 (5.5)	123 (5.7)	19 (6.0)	4 (3.1)
Tumor sidedness				
Right	1251 (55.1)	1188 (55.2)	154 (48.6)	66 (51.2)
Left	987 (43.4)	934 (43.4)	159 (50.2)	63 (48.8)
Unknown	33 (1.5)	30 (1.4)	4 (1.3)	
Degree of differentiation				
High	145 (6.4)	138 (6.4)	19 (6.0)	10 (7.8)
Middle	1574 (69.3)	1504 (69.9)	228 (71.9)	76 (58.9)
Poor	346 (15.2)	314 (14.6)	45 (14.2)	34 (26.4)
Unknown	205 (9.1)	196 (9.1)	25 (7.9)	9 (7.0)
Chemotherapy	129 (5.7)	NA	25 (7.9)	129 (100.0)

Data are presented as means (± SD) or numbers (%)

*NA* not applicable

^a^This population was used to estimate a time-independent hazard ratio for the transition DIAG-90DM

^b^Patients who underwent surgery and did not receive adjuvant chemotherapy. This population was used to estimate the transitions DIAG-DOC and DIAG-REC

^c^Patients who underwent surgery and developed a recurrence, independent of adjuvant chemotherapy. This population was used to estimate the transition REC-DEATH

^d^Note that this subpopulation of adjuvant treated patients was only used for external validation of the PATTERN model and not for model parametrization

### Model-based predictions for three selection strategies

We illustrated the application of our model by evaluating the health gain of the following selection strategies: (A) 100% adherence to the 2018 NVMO guideline, (B) observed adherence to NVMO guideline recommendations and (C) a biomarker-driven strategy. To explicitly demonstrate the impact of treatment on recurrence-free survival and colon cancer survival within 5 years, an additional strategy was evaluated: (D) none of the patients receive adjuvant chemotherapy.

In the 2018 NVMO guideline strategy, adjuvant chemotherapy is only considered in stage II colon cancer patients with pT4 tumors that are MSS. In the observed adherence to the NVMO guideline strategy, adherence was based on treatment allocation according to the most recent NCR data; that is 21% of the patients with a pT4 tumor and MSS receive chemotherapy and 4% of patients who do not meet these high-risk requirements. In the hypothetical biomarker-driven strategy, we assumed that all patients with MSS tumors in combination with a mutation in BRAF or KRAS receive adjuvant chemotherapy.

## Results

### Characteristics of the patient population

In Table 1, the baseline characteristics for the NCR cohort are shown for the whole population (*n* = 2271), subpopulation 1 (*n* = 2152) and subpopulation 2 (*n* = 317). In the whole NCR cohort, the majority of patients was aged > 70 (59.6%), had a pT3 stage tumor (88.4%), less than 10 lymph nodes examined (52.8%) and a right-sided tumor (55.1%). 344 recurrences and 751 deaths were observed. Follow-up duration of the patients was at least 36 months, with a maximum of 179 months. The median follow-up duration was 53 months. There were no missing values in the follow-up measurements and age. Only 5.5% of the patients had missing values in one of the clinical features (pT stage, evaluated lymph nodes and tumor sidedness). These missing values were not related to the follow-up measurements.

Baseline characteristics for the MATCH cohort, Texas cohort and DONOSTIA cohort are shown in Table 2. In the MATCH cohort the average age was 69.9 years. The majority of the patients had a MSSdwt biomarker status (37.1%), followed by MSSmut (34.3%) and MSI (26.7%). In the Texas cohort, the average age was 69.9 years as well. The majority of patients had MSS status (51.9%), MSI status was present in 6.8% of the cases, for the remaining 41.4%, MMR status was unknown. In the DONOSTIA cohort, the average age was 69.1 and the majority of patients had MSS status (76.0%). For the three cohorts, 53 recurrences were observed within 5 years of follow-up. The median follow-up duration of the patients was 73 months, with a minimum of 2 months and a maximum of 290 months. There were no missing values in the follow-up measurements for all three cohorts.

**Table 2:** Patient characteristics of the MATCH cohort, Texas cohort and Donostia cohort

Variable	Whole population (*n* = 334)	MATCH cohort (*n* = 105)	Texas cohort (*n* = 133)	Donostia cohort (*n* = 96)
Age (years)	69.8 (10.6)	69.6 (7.8)	69.6 (11.9)	69.1 (11.9)
Gender				
Male	153 (45.8)	51 (48.6)	72 (54.1)	30 (31.3)
Female	181 (54.2)	54 (51.4)	61 (45.9)	66 (68.7)
MMR status				
MSI	60 (18.0)	28 (26.7)	9 (6.8)	23 (24.0)
MSS	219 (65.6)	77 (73.3)	69 (51.9)	73 (76.0)
Unknown	55 (16.4)	0 (0)	55 (41.4)	0 (0)
BRAF status				
Wild type	NA	90 (85.7)	NA	NA
Mutation	NA	13 (12.9)	NA	NA
Unknown	NA	2 (1.9)	NA	NA
KRAS status				
Wild type	NA	66 (62.9)	NA	NA
Mutation	NA	39 (37.1)	NA	NA
Unknown	NA	0 (0)	NA	NA
Biomarker subgroup				
MSI	60 (18.0)	28 (26.7)	9 (6.8)	23 (24.0)
MSSdwt	NA	39 (37.1)	NA	NA
MSSmut	NA	36 (34.3)	NA	NA
Unknown	55 (16.4)	2 (1.9)	55 (41.4)	0 (0)

Data are presented as means (± SD) or numbers (%)

*NA* not applicable, *MSI* microsatellite instability independent of status for *BRAF* and *KRAS*, *MSSdwt* microsatellite stability without a mutation for *BRAF* or *KRAS*; *MSSmut* microsatellite stability in combination with a mutation in *BRAF* or *KRAS*

### Estimates for transitions in the model with clinical and pathological features only

#### Transitions DIAG-90DM, DIAG-DOC, DIAG-REC

For the transition DIAG-90DM, we assumed a constant transition probability for the first three cycles. For the transition DIAG-DOC, a Gompertz distribution was fitted to estimate time to DOC (Table 3). Age was included as covariate in the survival model. For the transition DIAG-REC, we used a Gompertz distribution to estimate time to recurrence. The included covariates for the transition DIAG-REC are the number of evaluated lymph nodes, pT stage and tumor sidedness (Table 3). Patients with a missing value in one of the covariates were excluded from the analysis (5.5%).

**Table 3:** Parameter estimates specifying transitions in the PATTERN model

	Transition diagnosis to 90DM (DIAG-90DM)	Transition diagnosis to DOC (DIAG-DOC)	Transition diagnosis to recurrence (DIAG-REC)	Transition recurrence to death (REC-DOC + REC-DCC)^e^	Transition recurrence to DOC (REC-DOC)
Subpopulation^a^	Whole NCR population	1	1	2	2
Parametric distribution	NA	Gompertz	Gompertz	Log logistic	NA
*C*-statistic	NA	0.74 (0.71–0.78)	0.61 (0.56–0.66)	0.64 (0.61–0.68)	NA

	Probability	Coefficient (95% CI)	*p* value	Coefficient (95% CI)	*p* value	Coefficient (95% CI)	*p* value	Probability
Shape (intercept)	NA	0.010 (0.009; 0.012)	< 0.01	− 0.016 (− 0.021; − 0.010)	< 0.01	1.17 (1.06; 1.29)	< 0.01	NA
Rate/scale	NA	0.000 (0.000; 0.001)	0.02	0.004 (0.003; 0.005)	< 0.01	1,390 (397; 4,850)	0.48	NA
Age				NA	NA			
≤ 54	0.028 (0.004; 0.052)	Reference	< 0.01			− 3.439 (− 4.363; − 2.515)	< 0.01	0.000
55–59	0.028 (0.004; 0.052)	0.632 (− 0,045; 1,308)				− 3.766 (− 4.778; − 2.754)		0.001
60–64	0.032 (0.011; 0.053)	0.591 (− 0.037; 1,219)				− 4.094 (− 5.194; − 2.994)		0.001
65–69	0.032 (0.011; 0.053)	1.039 (0.453; 1.624)				− 4.421 (− 5.609; − 3.233)		0.001
70–74	0.064 (0.040; 0.087)	1.701 (1.150; 2.252)				− 4.749 (− 6.025; − 3.473)		0.002
75–79	0.073 (0.050; 0.097)	2.159 (1.617; 2.702)				− 5.076 (− 6.440; − 3.712)		0.003
80–84	0.114 (0.081; 0.147)	2.823 (2.281; 3.365)				− 5.404 (− 6.856; − 3.952)		0.004
85–89	0.156 (0.099; 0.213)	3.166 (2.604; 3.729)				− 5.731 (− 7.271; − 4.191)		0.005
90–95	0.333 (0.178; 0.488)	3.315 (2623; 4.007)				− 6.059 (− 7.687; − 4.431)		0.006
Lymph nodes evaluated (≥ 10 vs < 10)	NA	NA	NA	− 0.519 (− 0.762; − 0.276)	< 0.01	NA	NA	NA
pT stage (pT4 vs pT3)	NA	NA	NA	1.081 (0.779; 1.383)	< 0.01	NA	NA	NA
Tumor sidedness (left vs right)	NA	NA	NA	0.505 (0.272; 0.737)	<0.01	NA	NA	NA
Biomarker subgroup	NA	NA	NA			NA	NA	NA
MSI				− 1.398^b^ (− 2.571; − 0.224)	0.23			
MSSdwt				− 0.128^b^ (− 2.652; 2.400)	0.45			
MSSmut				0.424^b^ (− 2.025; 2.876)	0.68			
Treatment effect	NA	NA	NA	− 0.250^b,c^ (− 0.383; − 0.118)	< 0.01	NA	NA	NA
	NA	NA	NA	− 0.063^b,d^ (− 0.276; 0.149)	0.13	NA	NA	NA

*90DM* 90-day mortality, *DOC* death other causes, *NA* not applicable

^a^Population on which the model is fitted

^b^Parameters used in the model are the log transformations of the estimated hazard ratios of 0.247, 0.880, 1.528, 0.779, 0.939 respectively for MSI, MSSdwt, MSSmut and treatment effect

^c^Treatment effect for fluoropyrimidine monotherapy compared to no adjuvant chemotherapy

^d^Treatment effect for FOLFOX compared to Fluoropyrimidine monotherapy

^e^ REC-DOC and REC-DCC were estimated in the same parametric survival model. Transition REC-DCC is calculated as the difference of transition REC-DOC + REC-DCC and transition REC-DOC

#### Transitions REC-DOC and REC-DCC.

For transitions REC-DOC and REC-DCC, we first used a Log logistic distribution to estimate time to death after a recurrence. Age was included as covariate in the survival model (Table 3). We subsequently differentiated between DOC and DCC, as described above. Tunnel states were used to correctly incorporate the time-dependent HR for transitions REC- DOC and REC-DCC in the model.

### Subgroups included in the PATTERN model

The transition probabilities are dependent on prognostic factors, the hazards to transit vary between patients based on their clinical and pathological factors. For that reason, 72 subgroups are distinguished in the practical implementation of the cohort model based on; age (50–95) in nine 5-year categories, number of lymph nodes evaluated (< 10 and ≥ 10), pT stage (pT3 and pT4) and tumor sidedness (left and right).

### Internal validity of the model with clinical and pathological features only

In Online Appendix Figs. 1–3, the ability of the simulation model to reproduce the data is shown for the transitions DIAG-DOC, DIAG-REC and REC-DEATH (REC to DOC + DCC) separately. For the transition DIAG-DOC, the predicted number of deaths fits well to the observed death rate (Online Appendix Fig. 1). For the transition DIAG-REC, the predicted number of recurrences is less close to the data. Especially, the predictions for the subgroups with a pT4 profile, for which the sample size was small, deviate from the observed data. However, all Kaplan–Meier curves lie within the 95% confidence interval of the parametric survival model (Online Appendix Fig. 2). Transitions REC-DOC and REC-DCC were estimated in one survival model. In contrast to the survival model used for the transition DIAG-DOC, age was added as a continuous covariate in this survival analysis because the small sample sizes of the subgroups for age hampered inclusion of age as a categorical variable. For the sum of the transitions REC-DOC and REC-DCC, mortality predictions fit in general well with the death rate in the dataset. In age categories 85–89 and 90–95, the model deviates from the data, probably due to the small sample size in these subgroups (Online Appendix Fig. 3).

Results of the Greenwood–D’Agostino–Nam test for model calibration are shown in Online Appendix Fig. 4. For all three transitions, DIAG-DOC, DIAG-REC and REC-DEATH, sufficient model calibration was shown (*p* values of 0.93, 0.16 and 0.29, respectively). In addition, *C*-statistics were 0.74 (0.71–0.78), 0.63 (0.58–0.68) and 0.64 (0.61–0.68) for the transitions DIAG-DOC, DIAG-REC and REC-DEATH, respectively, (Table 3), which indicate sufficient to good model discrimination [35]. Finally, the model predictions corresponded reasonably well with the NCR data used for model development; 73% (66 out of 90) of the number of time points for which, in this validation exercise, predictions were obtained in the general population and in the subgroup populations, were within the 95% CI of the data (Online Appendix Table 2).

### Addition of biomarker status

The model including clinical and pathological factors was extended using three biomarker categories, i.e., MSI, MSSdwt and MSSmut. We used a Gompertz distribution to estimate HRs for the 3 subgroups with which we could correct the transition DIAG-REC. HRs of 0.25 (0.08; 0.80), 0.88 (0.08; 11.02) and 1.53 (0.13; 17.75) were estimated for the MSI, MSSdwt and MSSmut subgroups, respectively. Parameters are shown in Table 3 and the ability of the simulation model to reproduce the data is shown in Online Appendix Fig. 5.

### Addition of treatment effect

We implemented treatment effect in the decision model by adjusting the transition DIAG-REC; the shape parameter of the Gompertz distribution was multiplied with the previously estimated HR of 0.73 [24].

### External validation of the PATTERN model

The results of the external validation of the PATTERN model are shown in Online Appendix Table 2. 72% (13 out of 18), 64% (23 out of 36) and 56% (30 out of 54) of the model predictions, in the general population and in the subgroup populations, fitted within the 95% CI of the 2015 NCR data at months 12, 24 and 36, respectively. In general, the model predictions fitted reasonably well with the 2015 data, except for the pT4 subgroup. In this subgroup, at none of the time points, the model-predicted recurrence rates fitted within the 95% confidence interval. Based on these findings and after discussion with clinical experts, the PATTERN model was updated to 2015 for the pT4 subgroup. The transition from recurrence to diagnosis is the only transition in the PATTERN model in which pT stage was included as covariate. We re-estimated the transition DIAG-REC in the NCR 2015 data and compared the betas for pT stage to the estimates in the 2002–2008 NCR data. The beta in the 2015 NCR data was 1.47, which is a factor 1.36 higher compared to the 2002–2008 NCR data. The transition from diagnosis to recurrence in the PATTERN model was, therefore, adjusted by multiplying the original beta for pT stage in the PATTERN model by the factor 1.36. After the model update, the external validation was repeated and showed that 83%, 81% and 70% of the model predictions fitted within the 95% CI of the data at months 12, 24 and 36, respectively. Especially, the fit for the pT4 subgroup improved (Online Appendix Table 2).

Validation of the treatment effect that was implemented in the PATTERN model based on external RCT data,showed that overall 94% of the model predictions for recurrence and overall survival fitted within the 95% confidence interval of the data (Online Appendix Table 4).

### Model-based predictions for the selection strategies

For the selection strategy in which none (strategy D) of the patients received adjuvant chemotherapy, the model predicted 126 recurrences and 89 deaths due to colon cancer after 5 years in a cohort of 1000 patients.

**Fig. 3:**
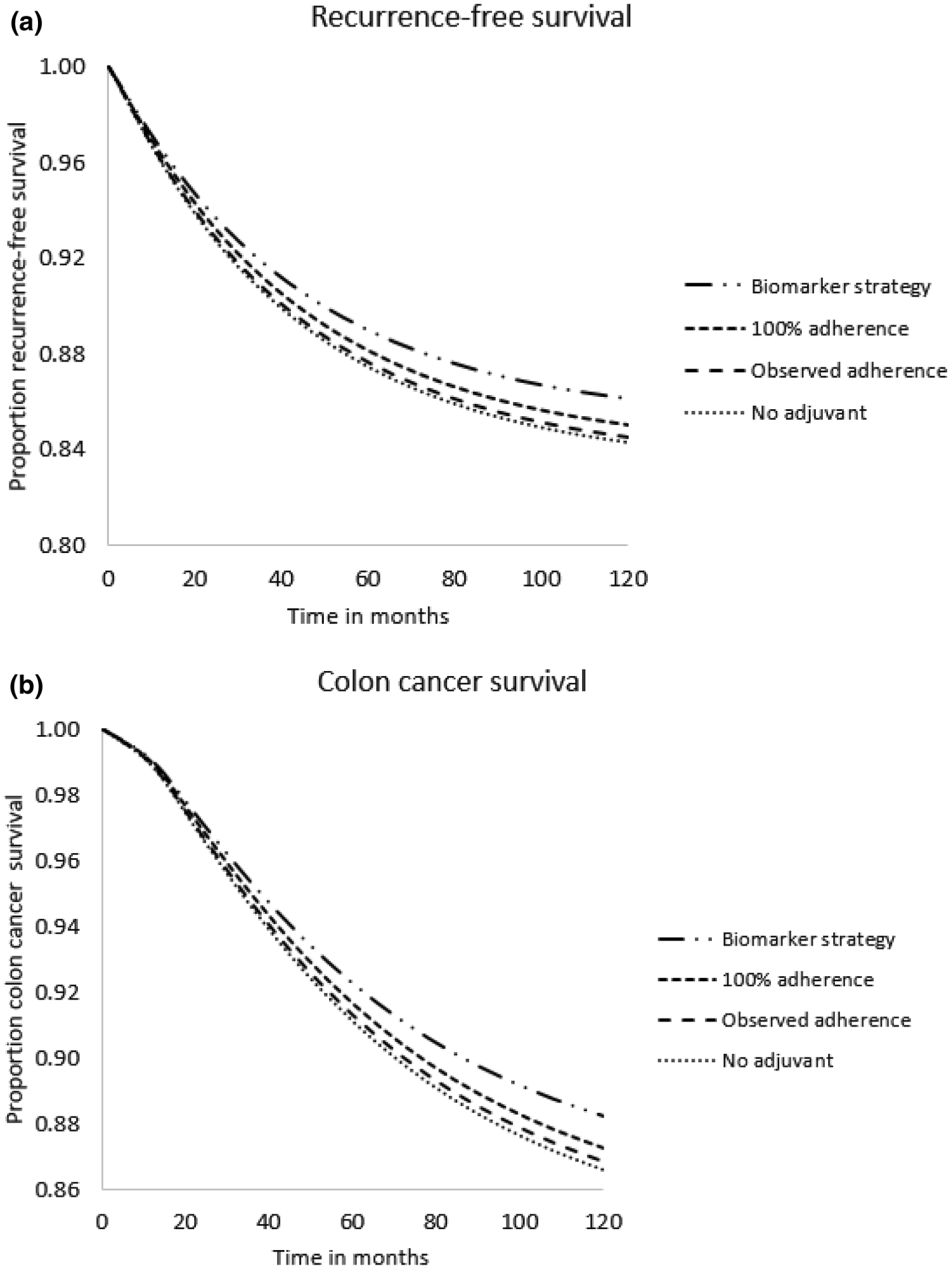
Model predictions for recurrence-free survival (**a**) and deaths due to colon cancer (**b**)

For the observed adherence to guideline recommendations strategy, the model predicted 123 recurrences after 5 years in a cohort of 1000 patients. For deaths due to colon cancer, this figure was 87. In case of 100% adherence to the 2018 NVMO guideline recommendations, the model predicted 119 recurrences and 83 deaths due to colon cancer in a 5-year time horizon. This is a decrease of 3.3% in the number of recurrences and a decrease of 4.6% in DCC compared to the observed adherence to guideline recommendations strategy. For the hypothetical biomarker-driven strategy, 110 recurrences and 77 deaths due colon cancer were predicted after 5 years. Compared to observed adherence to guideline recommendations, this is a decrease of 10.6% in the number of recurrences and a decrease of 11.5% in DCC. Model predictions are shown in Fig. 3.

## Discussion

In this study, a Markov cohort model was developed for the future population-level evaluation of different strategies to improve the selection of stage II colon cancer patients for adjuvant chemotherapy. The decision model describes the influence of pT stage, number of lymph nodes evaluated, tumor sidedness, MSS status, BRAF mutation status, and KRAS mutation status on relevant outcomes, such as the recurrence rate and disease-specific survival. Sufficiently adequate internal and external validity of the model was demonstrated. To illustrate the application of the model, we evaluated the potential health gain that can be achieved with 100% adherence to the 2018 NVMO guideline recommendations for adjuvant chemotherapy compared to observed adherence to these recommendations. A hypothetical biomarker selection strategy was evaluated as well. Full adherence to the NVMO guideline and the biomarker strategy resulted in a 4.6% and 11.5% decrease, respectively, in colon cancer mortality compared to observed adherence to NVMO recommendations.

A Markov decision model for the evaluation of adjuvant chemotherapy in stage II colon cancer was developed earlier by Avayci et al*.* [36]. These authors used the model to assess the cost-effectiveness of adjuvant treatment compared to no adjuvant treatment*.* This study concluded that fluorouracil monotherapy was cost-effective; whereas, fluorouracil in combination with oxaliplatin was considered as not cost-effective compared to no treatment. However, this model does not distinguish between different patient groups with a different prognosis, and is therefore not able to evaluate different selection strategies for adjuvant treatment. To our knowledge, the PATTERN model is the first model that can compare different strategies for selecting high-risk stage II colon cancer patients for adjuvant therapy. In addition, the PATTERN model is able to perform evaluations for both adjuvant treatment with fluoropyrimidine monotherapy and fluoropyrimidine combined with oxaliplatin.

To develop the PATTERN model, we assumed that all deaths during the first 90 days after diagnosis in the NCR dataset were caused by complications of surgery or poor clinical condition of the patient. The estimated probabilities of 0.029, 0.051 and 0.12 for age categories < 65, 65–74 and > 74, respectively, were in line with previously reported probabilities (0.022, 0.045 and 0.12, respectively) [23]. Furthermore, we assumed that the probability to die from DOC was slightly different in the NCR population compared to the general population, due to a different selection of individuals. To illustrate, the factors that increase the risk of developing colon cancer are also risk factors for developing other chronic diseases, such as cardiovascular diseases [37]. Therefore, we used the NCR data to estimate the transitions to DOC instead of life tables from the Central Bureau for Statistics Netherlands (CBS). As a check, we compared our estimates to the CBS, which showed that the probabilities to die from DOC as estimated in our data were higher for ages lower than 65 and were in line for ages 65–85. After age 85, the probability to die due to other causes was lower in our dataset compared to the CBS. A reason for the difference in probability to die from DOC in the population aged < 65 could be the different selection of individuals compared to the general population. The difference in patients aged > 85 might be explained due to the fact that not all people aged above the 85 are eligible for the initial surgery due to reduced clinical condition. As we only included patients with initial surgery in our analysis, we probably had a selection of patients aged above 85 with a better clinical condition compared to the general population. In addition, we also assumed that patients can only die from colon cancer after having a recurrence. That is, patients in our model cannot directly transit from diagnosis to DCC. As there may be some underreporting of the number of recurrences in the NCR data, it is possible that the probability to die from colon cancer was also underestimated in the decision model, which could result in an underestimation of the impact of adjuvant treatment.

In our dataset, 3-year and 5-year disease-free survival (DFS) without adjuvant treatment was 0.89 and 0.86, respectively. These probabilities were higher compared to those found in the literature. For example, in the QUASAR trial, probabilities of 0.81 and 0.76 were found for 3-year and 5-year DFS, respectively [38]. There are several reasons that may explain this difference. First, the populations are not completely comparable at baseline. The percentage of patients with a pT4 stage in QUASAR was 17.4% compared to 9.6% in our data. It should be noted that direct comparison of baseline characteristics is hampered due to the fact that the QUASAR trial population also included stage III patients (8%) and patients with rectal cancer (29%). Second, the patient inclusion of the QUASAR trial took place in 1994–2003, compared to diagnosis years 2002–2008 in the dataset used in the current study. Literature shows that quality of diagnosis has improved over the last decades [39]. Therefore, it is possible that stage III patients were classified as stage II patients in QUASAR, which has worsened the overall DFS for stage II patients in the QUASAR trial. Finally, we cannot exclude the possibility that recurrences may have been missed in the Netherlands cancer registry (NCR). Overall, the PATTERN model was quantified based on Dutch DFS and OS rates. As a consequence, our model predictions are only generalizable to countries with similar survival rates.

In the survival model that was used to calculate the transition probabilities for diagnosis to recurrence, the following factors were found to be prognostic: pT stage, tumor sidedness and number of evaluated lymph nodes. Degree of differentiation, despite previously included in the guidelines, was not a prognostic factor in our dataset. This finding is in line with Snaebjornsson et al. [40] reporting that there is no support to take poor differentiation as a high-risk factor in stage II colon cancer patients into account when deciding on the administration of adjuvant chemotherapy. Despite the fact that tumor sidedness is not included in the guidelines as a prognostic factor for recurrence, this factor is nevertheless included in our survival model because of its strong prognostic effect in favor of the right-sided tumors. This is in line with a population-based SEER analysis of 33,323 stage II colon cancer patients which demonstrated that both 5 year OS and DFS were superior in right-sided compared to left-sided colon cancers (HR 0.85, 95% CI 0.81; 0.89 and HR 0.75, 95% CI 0.70; 0.80, respectively) [41]. It should be noted that this is contradictory to the majority of studies regarding the prognostic value of primary tumor location, which indicated that patients with a right-sided tumor have in general a worse prognosis compared to left-sided tumors [42, 43]. Overall, the majority of the prognostic factors found in our data were in line with the literature [44].

The external validation, for which the 2015 NCR data were used, showed overall good agreement between the model predictions and the external data, except for the pT4 subgroup. The model underestimated the number of recurrences in this subgroup. The difference in recurrence rate in cohort 2002–2008 and cohort 2015 could potentially be explained by the increased awareness of the poorer prognosis of pT4 stage II patients compared to pT3 stage II patients, which was especially triggered by the published findings of the MOSAIC trial in 2004 [4]. It could be that as a result of these findings, pT4 patients are currently better monitored after diagnosis, leading to earlier detection of recurrences and, thus, higher recurrence rates. After discussion with clinical experts in the field, we decided to update the beta for pT stage, to inform the PATTERN model with the most recent information available.

Because there is currently a lack of knowledge in the field regarding the most (cost-)effective manner to assign treatment in stage II colon cancer patients, it is important to combine all the knowledge we have acquired over the past decade in a decision model to enable evaluation of selection strategies. It is expected that the number of patients with stage II colon cancer will increase due to the introduction of the Dutch CRC screening program, thereby increasing the importance of treating these patients optimally. The PATTERN model can address this issue, as has been demonstrated in our simulation of 3 hypothetical strategies for 100% adherence to the 2018 NVMO guidelines, observed adherence to guideline recommendations and a biomarker-driven strategy.

To adequately interpret the results of these simulations, a number of issues require attention. First, treatment heterogeneity was not included in the PATTERN model. In a previous study, a predictive effect was found for patients with a microsatellite instable (MSI) tumor. In addition to having a favorable prognosis, it was shown that these patients have a certain resistance to fluorouracil-based chemotherapy [17]. The predictive treatment effect for the MSI subgroup was not taken into account in the PATTERN model, because this patient subgroup is not eligible for adjuvant treatment in the current guideline because of its favorable prognosis. For the BRAF and KRAS biomarkers and the other included prognostic features in the model, no predictive effect for adjuvant chemotherapy has yet been demonstrated in stage II colon cancer patients. It should be noted that the PATTERN model was built in a flexible manner, e.g., treatment heterogeneity can be implemented when the required data become available. Second, it was assumed that the distribution of biomarker status was independent of clinical and pathological factors. There is no clear evidence in favor or against this assumption. Third, due to limitations in the data, no distinction was made in the MSI group for the presence or absence of a mutation in BRAF and/or KRAS. In addition, we did not correct for heterogeneity between the cohorts used for biomarker analysis.

Moreover, it should be noted that we did not aim to conduct a full cost-effectiveness analysis to determine the optimal selection strategy for patients with stage II colon cancer. Instead, we solely evaluated the impact on health gain of a limited number of example strategies on recurrence and death to illustrate the application of the model. In this example analysis, we did not include adverse effects of adjuvant chemotherapy on quality of life and costs. In order to evaluate selection strategies from a health economic perspective, the PATTERN model will be further informed with cost data and quality adjusted life years (QALYs) data.

To conclude, we presented the development of the Personalized Adjuvant TreaTment in EaRly stage coloN cancer (PATTERN) model which is, to our knowledge, the first model that allows a population-level comparison of different personalized strategies for selecting high-risk stage II colon cancer patients for adjuvant chemotherapy. The model includes clinical and pathological features as well as biomarker status for MSS, BRAF and KRAS. This model will be used to evaluate the effectiveness and cost-effectiveness of existing and biomarker-based selection strategies to improve treatment allocation in stage II colon cancer patients.

## Electronic supplementary material

Below is the link to the electronic supplementary material.

Supplementary file1 (DOCX 433 kb)

## Data Availability

The NCR registry data that were used for model development are available upon reasonable request from the Netherlands Cancer Registry.
