# Supplementary material for: Modeling Personalized Adjuvant TreaTment in EaRly stage coloN cancer (PATTERN)
Source: Eur J Health Econ. 2020 May 26;21(7):1059–73. doi: 10.1007/s10198-020-01199-4 (PMC7423797; doi:10.1007/s10198-020-01199-4)
Supplement: Supplementary file 1 — Supplementary file1 (DOCX 433 kb) [file 10198_2020_1199_MOESM1_ESM.docx]

**Appendix Table 1.** Patient characteristics of the 2015 NCR cohort, which was used for the external validation of the PATTERN model.

| **Variable** | **Non-adjuvant treated population**  **(n=1,214)** | **Adjuvant**  **treated population**  **(n=115)** |
| --- | --- | --- |
| Age (years) | 72.0 (9.5) | 62.6 (7.8) |
| Gender |  |  |
| Male | 627 (51.6) | 63 (54.8) |
| Female | 587 (48.4) | 52 (45.2) |
| pT stage |  |  |
| pT3 | 1,089 (89.7) | 60 (52.2) |
| pT4  Unknown | 124 (10.2)  1 (0.1) | 55 (47.8)  NA |
| Evaluated lymph nodes |  |  |
| <10 | 53 (4.4) | 8 (7.0) |
| ≥10 | 1,161 (95.6) | 107 (93.0) |
| Tumor site |  |  |
| Right | 714 (58.8) | 54 (47.0) |
| Left  Unknown | 481 (49.6)  19 (1.6) | 59 (53.0)  NA |
| Data are presented as means (±SD) or numbers (%). NA = not applicable. | | |

**Appendix Table 2**. Results of the internal validation (NCR cohort 2002-2008) and external validation (NCR cohort 2015). The results of the external validation are reported for both before and after the model update.

|  | Recurrence | | | | Overall survival | | | |
| --- | --- | --- | --- | --- | --- | --- | --- | --- |
|  | Data estimate | | Model prediction | | Data estimate | | Model prediction | |
|  | Point estimate | 95% CI | Before model update^a^ | After model update^a^ | Point estimate | 95% CI | Before model update^a^ | After model update^a^ |
| Overall population | | | | | | | | |
| 2002-2008 | n=2,211 | | | | n=2,206 | | | |
| 12 months | 0.95 | 0.94-0.96 | 0.95 | NA | 0.88 | 0.86-0.89 | 0.90 | NA |
| 24 months | 0.90 | 0.89-0.92 | 0.91 | NA | 0.81 | 0.80-0.83 | 0.84 | NA |
| 36 months | 0.87 | 0.85-0.89 | 0.89 | NA | 0.76 | 0.75-0.78 | 0.79 | NA |
| 48 months | 0.85 | 0.83-0.86 | 0.87 | NA | 0.72 | 0.70-0.74 | 0.73 | NA |
| 60 months | 0.83 | 0.82-0.85 | 0.85 | NA | 0.67 | 0.65-0.69 | 0.68 | NA |
| 2015 | n=1,214 | | | | n=1,214 | | | |
| 12 months | 0.96 | 0.94-0.97 | 0.96 | 0.96 | 0.92 | 0.91-0.93 | 0.90 | 0.90 |
| 24 months | 0.90 | 0.89-0.92 | 0.93 | 0.92 | 0.88 | 0.86-0.89 | 0.84 | 0.85 |
| 36 months | 0.87 | 0.84-0.89 | 0.91 | 0.90 | 0.83 | 0.82-0.84 | 0.79 | 0.79 |
| pT3 population | | | | | | | | |
| 2002-2008 | n=1,979 | | | | n=1,974 | | | |
| 12 months | 0.97 | 0.95-0.97 | 0.96 | NA | 0.90 | 0.89-0.92 | 0.90 | NA |
| 24 months | 0.92 | 0.90-0.93 | 0.93 | NA | 0.85 | 0.83-0.86 | 0.85 | NA |
| 36 months | 0.89 | 0.87-0.90 | 0.90 | NA | 0.80 | 0.78-0.81 | 0.80 | NA |
| 48 months | 0.86 | 0.84-0.88 | 0.88 | NA | 0.75 | 0.73-0.77 | 0.74 | NA |
| 60 months | 0.85 | 0.83-0.87 | 0.87 | NA | 0.71 | 0.69-0.73 | 0.69 | NA |
| 2015 | n=1,089 | | | | n=1,089 | | | |
| 12 months | 0.97 | 0.96-0.98 | 0.97 | 0.97 | 0.95 | 0.94-0.97 | 0.91 | 0.91 |
| 24 months | 0.93 | 0.91-0.94 | 0.94 | 0.94 | 0.91 | 0.89-0.93 | 0.86 | 0.86 |
| 36 months | 0.90 | 0.87-0.91 | 0.93 | 0.93 | 0.88 | 0.86-0.89 | 0.81 | 0.81 |
| pT4 population | | | | | | | | |
| 2002-2008 | n=179 | | | | n=179 | | | |
| 12 months | 0.84 | 0.78-0.89 | 0.89 | NA | 0.79 | 0.73-0.85 | 0.89 | NA |
| 24 months | 0.74 | 0.67-0.81 | 0.81 | NA | 0.65 | 0.58-0.72 | 0.80 | NA |
| 36 months | 0.70 | 0.62-0.77 | 0.75 | NA | 0.61 | 0.53-0.68 | 0.72 | NA |
| 48 months | 0.67 | 0.59-0.75 | 0.71 | NA | 0.52 | 0.45-0.60 | 0.65 | NA |
| 60 months | 0.64 | 0.56-0.72 | 0.68 | NA | 0.48 | 0.41-0.56 | 0.59 | NA |
| 2015 | n=124 | | | | n=124 | | | |
| 12 months | 0.81 | 0.73-0.89 | 0.91 | 0.87 | 0.85 | 0.80-0.92 | 0.86 | 0.85 |
| 24 months | 0.67 | 0.58-0.77 | 0.85 | 0.77 | 0.74 | 0.67-0.89 | 0.77 | 0.74 |
| 36 months | 0.61 | 0.51-0.71 | 0.80 | 0.71 | 0.68 | 0.57-0.80 | 0.69 | 0.64 |
|  | Aged <70 | | | | | | | |
| 2002-2008 | n=894 | | | | n=891 | | | |
| 12 months | 0.95 | 0.93-0.96 | 0.95 | NA | 0.95 | 0.93-0.96 | 0.96 | NA |
| 24 months | 0.90 | 0.88-0.92 | 0.91 | NA | 0.91 | 0.89-0.93 | 0.93 | NA |
| 36 months | 0.87 | 0.85-0.89 | 0.88 | NA | 0.87 | 0.85-0.89 | 0.90 | NA |
| 48 months | 0.85 | 0.82-0.87 | 0.85 | NA | 0.84 | 0.81-0.86 | 0.87 | NA |
| 60 months | 0.84 | 0.81-0.86 | 0.84 | NA | 0.80 | 0.78-0.83 | 0.83 | NA |
| 2015 | n=517 | | | | n=517 | | | |
| 12 months | 0.96 | 0.94-0.98 | 0.96 | 0.96 | 0.97 | 0.95-0.98 | 0.96 | 0.96 |
| 24 months | 0.91 | 0.89-0.94 | 0.93 | 0.93 | 0.95 | 0.92-0.98 | 0.93 | 0.93 |
| 36 months | 0.88 | 0.85-0.91 | 0.91 | 0.90 | 0.92 | 0.89-0.95 | 0.90 | 0.90 |
| Aged ≥70 | | | | | | | | |
| 2002-2008 | n=1,317 | | | | n=1,315 | | | |
| 12 months | 0.96 | 0.95-0.97 | 0.95 | NA | 0.83 | 0.81-0.85 | 0.86 | NA |
| 24 months | 0.90 | 0.89-0.92 | 0.92 | NA | 0.75 | 0.72-0.77 | 0.79 | NA |
| 36 months | 0.87 | 0.85-0.89 | 0.89 | NA | 0.69 | 0.66-0.71 | 0.72 | NA |
| 48 months | 0.84 | 0.82-0.87 | 0.87 | NA | 0.64 | 0.61-0.66 | 0.65 | NA |
| 60 months | 0.83 | 0.81-0.85 | 0.86 | NA | 0.58 | 0.56-0.61 | 0.58 | NA |
| 2015 | n=697 | | | | n=697 | | | |
| 12 months | 0.95 | 0.93-0.97 | 0.96 | 0.96 | 0.89 | 0.87-0.91 | 0.86 | 0.87 |
| 24 months | 0.89 | 0.87-0.92 | 0.94 | 0.93 | 0.82 | 0.79-0.85 | 0.79 | 0.79 |
| 36 months | 0.85 | 0.82-0.88 | 0.90 | 0.90 | 0.79 | 0.75-0.82 | 0.72 | 0.71 |
| <10 lymph nodes evaluated | | | | | | | | |
| 2002-2008 | n=1,151 | | | | n=1,148 | | | |
| 12 months | 0.94 | 0.93-0.95 | 0.94 | NA | 0.84 | 0.82-0.86 | 0.89 | NA |
| 24 months | 0.88 | 0.86-0.90 | 0.90 | NA | 0.77 | 0.74-0.79 | 0.83 | NA |
| 36 months | 0.83 | 0.81-0.85 | 0.86 | NA | 0.70 | 0.67-0.73 | 0.77 | NA |
| 48 months | 0.80 | 0.77-0.82 | 0.84 | NA | 0.65 | 0.62-0.68 | 0.71 | NA |
| 60 months | 0.79 | 0.77-0.83 | 0.82 | NA | 0.60 | 0.57-0.63 | 0.66 | NA |
| 2015 | n=53 | | | | n=53 | | | |
| 12 months | 0.91 | 0.83-0.99 | 0.93 | 0.92 | 0.81 | 0.71-0.92 | 0.88 | 0.88 |
| 24 months | 0.75 | 0.62-0.88 | 0.88 | 0.86 | 0.77 | 0.66-0.89 | 0.81 | 0.80 |
| 36 months | 0.72 | 0.59-0.86 | 0.84 | 0.82 | 0.67 | 0.56-0.78 | 0.74 | 0.73 |
| ≥ 10 lymph nodes evaluated | | | | | | | | |
| 2002-2008 | n=937 | | | | n=935 | | | |
| 12 months | 0.97 | 0.96-0.98 | 0.97 | NA | 0.92 | 0.90-0.94 | 0.91 | NA |
| 24 months | 0.93 | 0.92-0.95 | 0.94 | NA | 0.87 | 0.85-0.89 | 0.86 | NA |
| 36 months | 0.92 | 0.90-0.93 | 0.92 | NA | 0.84 | 0.81-0.86 | 0.81 | NA |
| 48 months | 0.90 | 0.88-0.92 | 0.90 | NA | 0.79 | 0.77-0.82 | 0.76 | NA |
| 60 months | 0.88 | 0.86-0.90 | 0.89 | NA | 0.76 | 0.73-0.78 | 0.72 | NA |
| 2015 | n=1,161 | | | | n=1,161 | | | |
| 12 months | 0.96 | 0.95-0.97 | 0.97 | 0.96 | 0.94 | 0.93-0.96 | 0.90 | 0.90 |
| 24 months | 0.91 | 0.89-0.93 | 0.94 | 0.93 | 0.90 | 0.89-0.93 | 0.85 | 0.85 |
| 36 months | 0.87 | 0.85-0.89 | 0.92 | 0.91 | 0.86 | 0.84-0.88 | 0.80 | 0.80 |
| Right sided tumor | | | | | | | | |
| 2002-2008 | n=1,222 | | | | n=1,218 | | | |
| 12 months | 0.96 | 0.94-0.97 | 0.96 | NA | 0.88 | 0.86-0.89 | 0.90 | NA |
| 24 months | 0.92 | 0.90-0.93 | 0.93 | NA | 0.83 | 0.80-0.85 | 0.84 | NA |
| 36 months | 0.89 | 0.87-0.91 | 0.91 | NA | 0.78 | 0.75-0.80 | 0.79 | NA |
| 48 months | 0.88 | 0.86-0.90 | 0.89 | NA | 0.74 | 0.72-0.77 | 0.74 | NA |
| 60 months | 0.87 | 0.85-0.89 | 0.88 | NA | 0.70 | 0.68-0.73 | 0.69 | NA |
| 2015 | n=714 | | | | n=714 | | | |
| 12 months | 0.96 | 0.95-0.98 | 0.97 | 0.97 | 0.92 | 0.88-0.94 | 0.89 | 0.89 |
| 24 months | 0.92 | 0.90-0.94 | 0.95 | 0.94 | 0.87 | 0.83-0.90 | 0.84 | 0.84 |
| 36 months | 0.88 | 0.86-0.91 | 0.93 | 0.93 | 0.82 | 0.79-0.85 | 0.79 | 0.80 |
| Left sided tumor | | | | | | | | |
| 2002-2008 | n=957 | | | | n=956 | | | |
| 12 months | 0.95 | 0.94-0.96 | 0.94 | NA | 0.88 | 0.86-0.90 | 0.90 | NA |
| 24 months | 0.88 | 0.86-0.91 | 0.89 | NA | 0.80 | 0.78-0.83 | 0.84 | NA |
| 36 months | 0.84 | 0.81-0.87 | 0.85 | NA | 0.75 | 0.72-0.77 | 0.78 | NA |
| 48 months | 0.80 | 0.77-0.83 | 0.82 | NA | 0.69 | 0.66-0.72 | 0.72 | NA |
| 60 months | 0.79 | 0.76-0.82 | 0.80 | NA | 0.64 | 0.61-0.67 | 0.67 | NA |
| 2015 | n=481 | | | | n=481 | | | |
| 12 months | 0.95 | 0.92-0.97 | 0.95 | 0.95 | 0.94 | 0.92-0.95 | 0.91 | 0.91 |
| 24 months | 0.88 | 0.85-0.91 | 0.91 | 0.91 | 0.89 | 0.86-0.92 | 0.86 | 0.86 |
| 36 months | 0.84 | 0.80-0.88 | 0.88 | 0.88 | 0.85 | 0.82-0.88 | 0.80 | 0.80 |
| Abbreviations: CI = confidence interval, NA = not applicable. ^a^ Based on the results of the external validation on the NCR 2015 data, a model update was needed. The external validation on the NCR 2015 data was repeated after model update. | | | | | | | | |

**Appendix Table 3.** Parameter estimates for the transition from diagnosis to recurrence for cohort 2002-2008 and cohort 2015.

|  | NCR cohort 2002-2008 | | | NCR cohort 2015 | | |
| --- | --- | --- | --- | --- | --- | --- |
|  | Estimate | 95% confidence interval | | Estimate | 95% confidence interval | |
|  |  | Lower | Upper |  | Lower | Upper |
| Shape | -0.016 | -0.021 | 0.010 | -0.005 | -0.017 | 0.007 |
| Rate | 0.004 | 0.003 | 0.005 | 0.006 | 0.003 | 0.012 |
| pT stage (pT4 vs pT3) | **1.081*** | 0.779 | 1.383 | **1.468*** | 1.098 | 1.838 |
| Lymph nodes evaluated (>10 vs ≤10) | -0.519 | -0.762 | -0.276 | -0.823 | -1.395 | -0.251 |
| Tumor side (Left vs Right) | 0.505 | 0.272 | 0.737 | 0.269 | -0.054 | 0.591 |
| Abbreviations: NCR = Netherlands Cancer Registry. *Note that the parameters in bold were used to determine the factor of 1.36, which was used to update the PATTERN model. | | | | | | |

**Appendix Table 4.** Results of the validation of treatment effect in the PATTERN model, which was implemented based on external Randomized Clinical Trial data. Model predictions under treatment with adjuvant chemotherapy for populations with characteristics conform the 2002-2008 NCR cohort and the 2015 NCR cohort, respectively, were compared to the observed survival curves of adjuvantly treated patients in these cohorts.

|  | Recurrence | | | | Overall survival | | | |
| --- | --- | --- | --- | --- | --- | --- | --- | --- |
|  | Data estimate | | Model prediction | | Data estimate | | Model prediction | |
|  | Point estimate | 95% CI | Before model update^a^ | After model  update^a^ | Point estimate | 95% CI | Before model update^a^ | After model update^a^ |
| Overall population | | | | | | | | |
| 2002-2008 | n=129 | | | | n=129 | | | |
| 12 months | 0.95 | 0.89-1.00 | 0.95 | NA | 0.96 | 0.93-0.99 | 0.95 | NA |
| 24 months | 0.83 | 0.74-0.91 | 0.91 | NA | 0.88 | 0.83-0.94 | 0.91 | NA |
| 36 months | 0.78 | 0.68-0.87 | 0.88 | NA | 0.84 | 0.77-0.90 | 0.88 | NA |
| 48 months | 0.77 | 0.67-0.86 | 0.85 | NA | 0.78 | 0.71-0.85 | 0.84 | NA |
| 60 months | 0.73 | 0.63-0.84 | 0.83 | NA | 0.76 | 0.68-0.83 | 0.80 | NA |
| 2015 | n=115 | | | | n=115 | | | |
| 12 months | 0.97 | 0.93-1.00 | 0.95 | 0.94 | 0.95 | 0.92-0.99 | 0.95 | 0.94 |
| 24 months | 0.88 | 0.81-0.94 | 0.91 | 0.89 | 0.90 | 0.84-0.96 | 0.91 | 0.91 |
| 36 months | 0.86 | 0.79-0.92 | 0.88 | 0.85 | 0.87 | 0.81-0.94 | 0.88 | 0.87 |
| Abbreviations: CI = confidence interval. ^a^ Based on the results of the external validation reported in Table 2, a model update was necessary. Therefore, validation of the implemented treatment effect in the PATTERN model was conducted before and after model update for cohort 2015. | | | | | | | | |


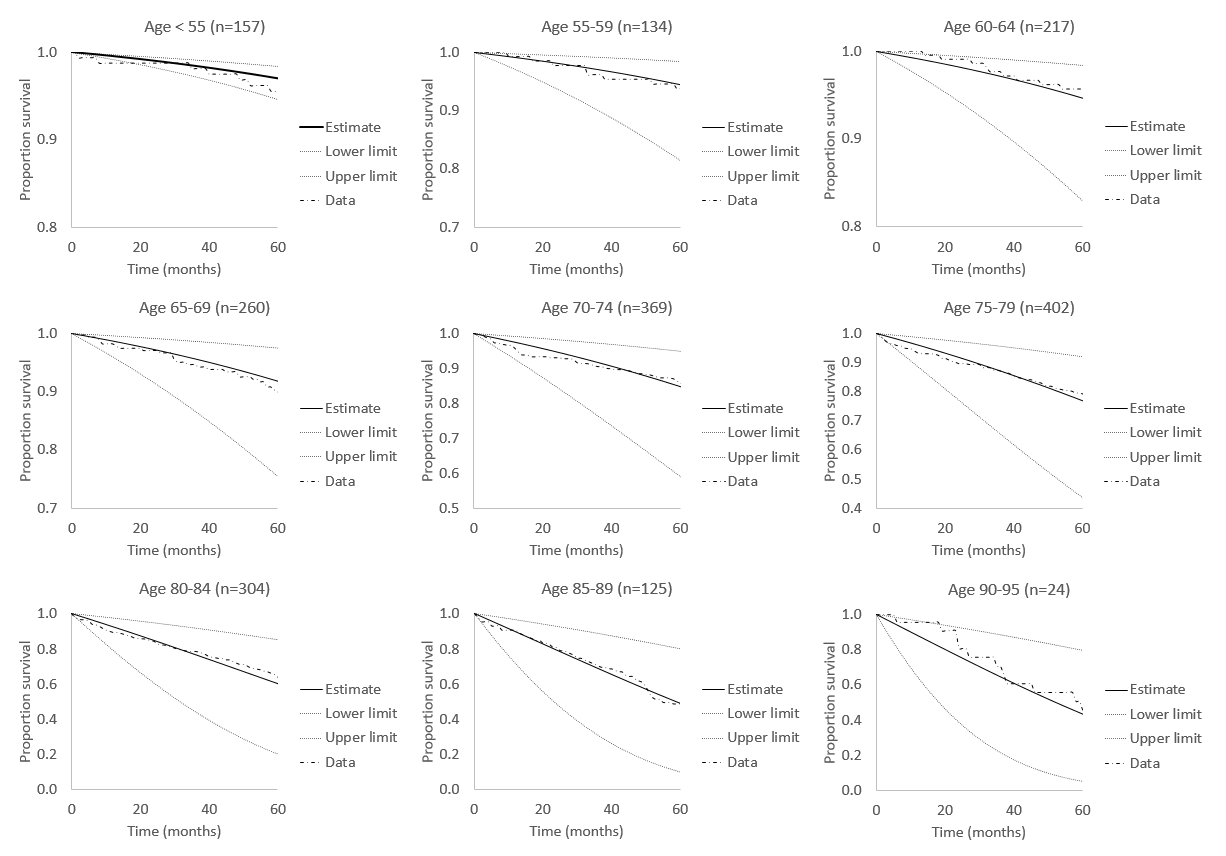


**Appendix Figure 1.** Parametric survival models for parametrization of the transition from diagnosis to death other causes. Each subpanel shows the data and the corresponding predicted survival in each age group including the lower and upper bound of the 95% confidence interval of the prediction. Note the different scaling for each subgroup on the y axis.


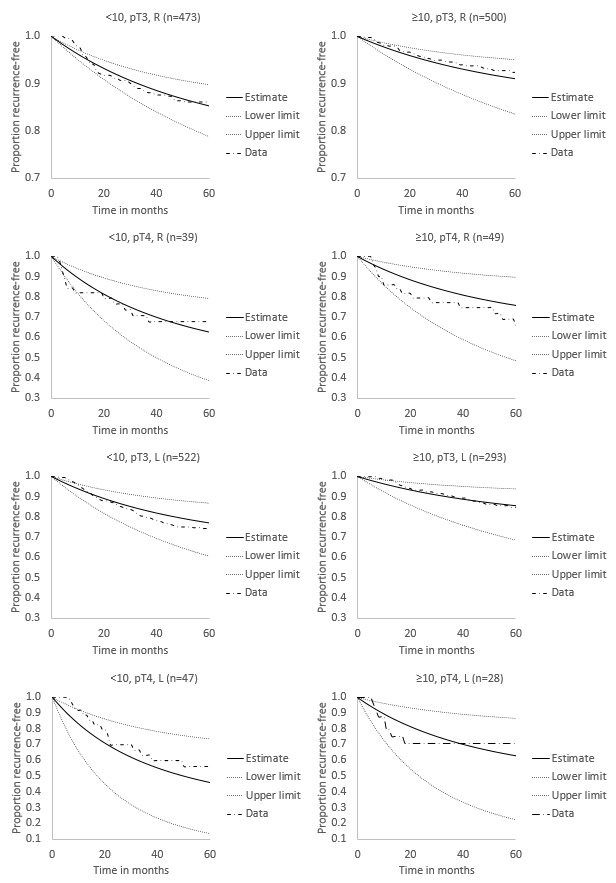


**Appendix Figure 2.** Parametric survival models for parametrization of the transition from diagnosis to recurrence. Each subpanel shows the data and the corresponding predicted recurrence rate in each subgroup including the lower and upper bound of the 95% confidence interval of the prediction. Note the different scaling for each subgroup on the y axis. Abbreviations: <10 = less than 10 lymph nodes evaluated; ≥ 10 = 10 or more lymph nodes evaluated; R = right sided tumor; L = left sided tumor.


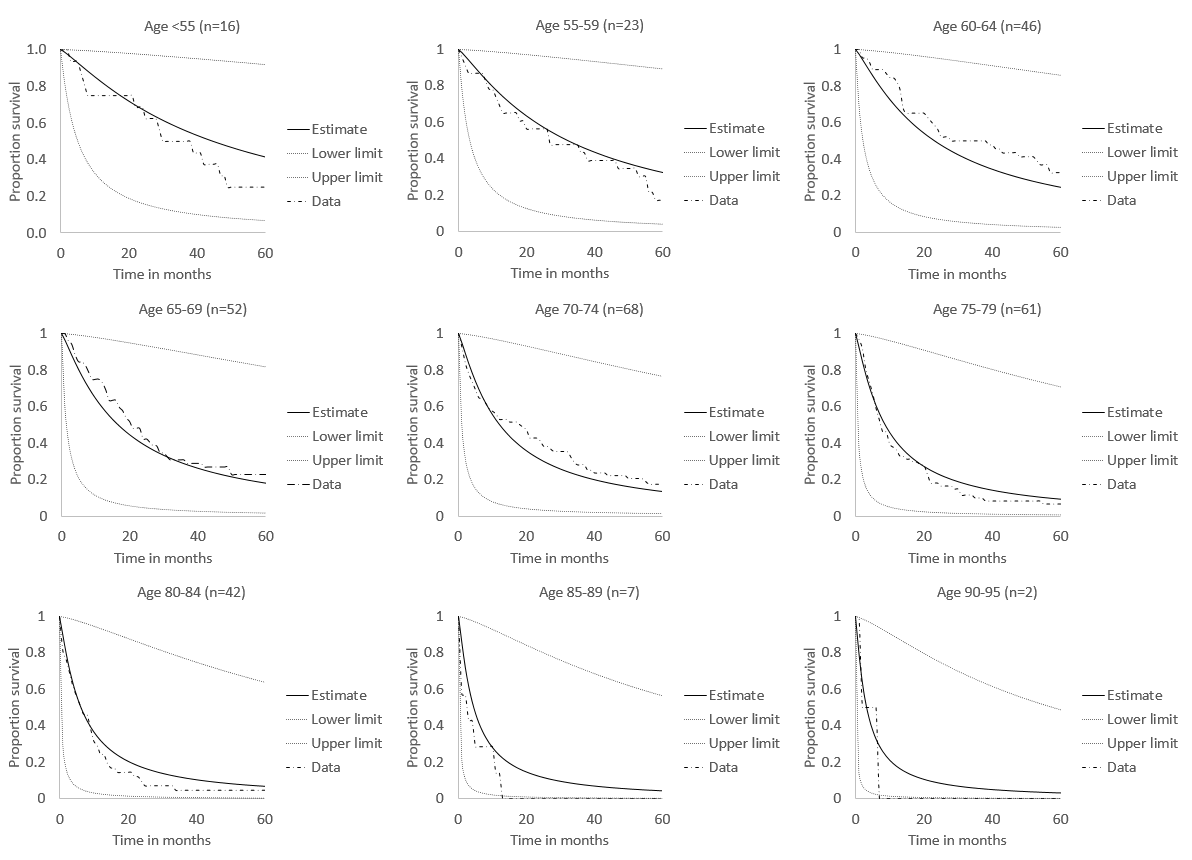


**Appendix Figure 3.** Parametric survival models for parametrization of the sum of the transitions from recurrence to death other causes and recurrence to death due to colon cancer. Each subpanel shows the data and the corresponding predicted survival in each age group including the lower and upper bound of the 95% confidence interval of the prediction.


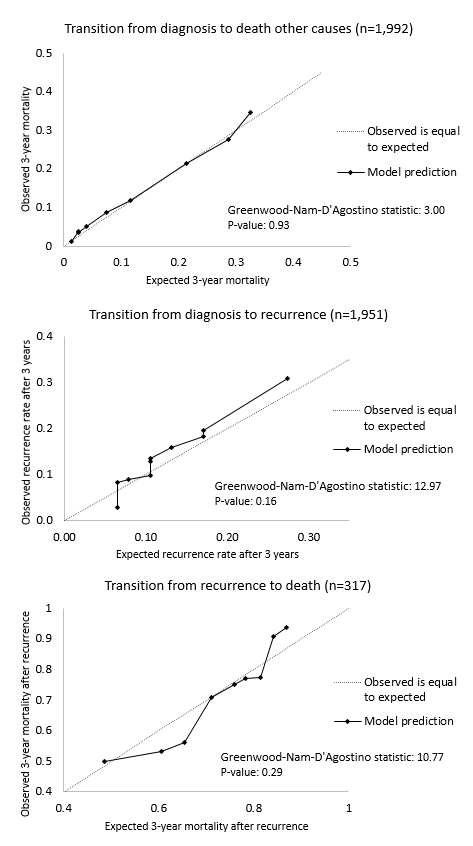
 (a)

(b)

(c)

**Appendix Figure 4.** Calibration plot for the parametric survival models for the transitions from diagnosis to death other causes (a), diagnosis tot recurrence (b) and recurrence to death (c).


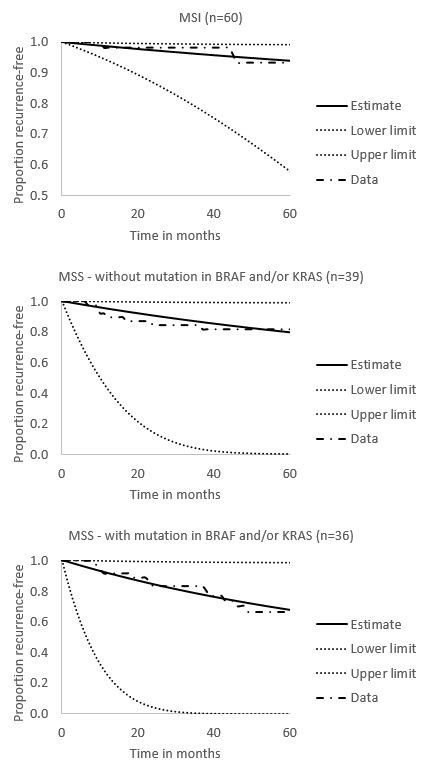
 **Appendix Figure 5.** Parametric survival models for the biomarker subgroups Microsatellite instability (MSI), Microsatellite stability without a mutation in BRAF and/or KRAS (MSSdwt) and Microsatellite stable in combination with a mutation in BRAF and/or KRAS (MSSmut). Each subpanel shows the data and the corresponding predicted recurrence-free survival in each biomarker group including the lower and upper bound of the 95% confidence interval of the prediction.
